# Supplementary material for: Pyruvate Dehydrogenase Kinase 1 inhibition mediated oxidative phosphorylation enhancement in cartilage promotes osteoarthritis progression
Source: BMC Musculoskelet Disord. 2023 Jul 20;24:597. doi: 10.1186/s12891-023-06585-6 (PMC10357736; doi:10.1186/s12891-023-06585-6)
Supplement: Supplementary file 1 — Additional file 1. [file 12891_2023_6585_MOESM1_ESM.zip › KEGG.Gsea.1653622432464/KEGG_PURINE_METABOLISM.html]

Details for gene set KEGG\_PURINE\_METABOLISM[GSEA]

|  || Dataset | 55457\_collapsed\_to\_symbols.cls.cls #OA\_versus\_normal.cls.cls #OA\_versus\_normal\_repos |
| Phenotype | cls.cls#OA\_versus\_normal\_repos |
| Upregulated in class | OA |
| GeneSet | KEGG\_PURINE\_METABOLISM |
| Enrichment Score (ES) | 0.3111094 |
| Normalized Enrichment Score (NES) | 1.5292218 |
| Nominal p-value | 0.0060790274 |
| FDR q-value | 0.053655177 |
| FWER p-Value | 0.742 |
Table: GSEA Results Summary

  

Fig 1: Enrichment plot: KEGG\_PURINE\_METABOLISM      
 Profile of the Running ES Score & Positions of GeneSet Members on the Rank Ordered List

  

| SYMBOL | TITLE | RANK IN GENE LIST | RANK METRIC SCORE | RUNNING ES | CORE ENRICHMENT || 1 | DGUOK | deoxyguanosine kinase [Source:HGNC Symbol;Acc:HGNC:2858] | 96 | 0.917 | 0.0114 | Yes |
| 2 | ADK | adenosine kinase [Source:HGNC Symbol;Acc:HGNC:257] | 127 | 0.878 | 0.0273 | Yes |
| 3 | PDE6B | phosphodiesterase 6B [Source:HGNC Symbol;Acc:HGNC:8786] | 172 | 0.838 | 0.0411 | Yes |
| 4 | NME7 | NME/NM23 family member 7 [Source:HGNC Symbol;Acc:HGNC:20461] | 178 | 0.835 | 0.0580 | Yes |
| 5 | POLR2G | RNA polymerase II subunit G [Source:HGNC Symbol;Acc:HGNC:9194] | 183 | 0.830 | 0.0749 | Yes |
| 6 | GMPR2 | guanosine monophosphate reductase 2 [Source:HGNC Symbol;Acc:HGNC:4377] | 253 | 0.783 | 0.0856 | Yes |
| 7 | POLR2J | RNA polymerase II subunit J [Source:HGNC Symbol;Acc:HGNC:9197] | 259 | 0.779 | 0.1013 | Yes |
| 8 | POLR2L | "RNA polymerase II, I and III subunit L [Source:HGNC Symbol;Acc:HGNC:9199]" | 333 | 0.733 | 0.1108 | Yes |
| 9 | NME5 | NME/NM23 family member 5 [Source:HGNC Symbol;Acc:HGNC:7853] | 362 | 0.720 | 0.1235 | Yes |
| 10 | ATIC | 5-aminoimidazole-4-carboxamide ribonucleotide formyltransferase/IMP cyclohydrolase [Source:HGNC Symbol;Acc:HGNC:794] | 401 | 0.706 | 0.1351 | Yes |
| 11 | AK1 | adenylate kinase 1 [Source:HGNC Symbol;Acc:HGNC:361] | 481 | 0.667 | 0.1427 | Yes |
| 12 | PAPSS2 | 3'-phosphoadenosine 5'-phosphosulfate synthase 2 [Source:HGNC Symbol;Acc:HGNC:8604] | 592 | 0.627 | 0.1470 | Yes |
| 13 | POLR2I | RNA polymerase II subunit I [Source:HGNC Symbol;Acc:HGNC:9196] | 640 | 0.609 | 0.1560 | Yes |
| 14 | POLR3K | RNA polymerase III subunit K [Source:HGNC Symbol;Acc:HGNC:14121] | 651 | 0.605 | 0.1677 | Yes |
| 15 | NME4 | NME/NM23 nucleoside diphosphate kinase 4 [Source:HGNC Symbol;Acc:HGNC:7852] | 677 | 0.599 | 0.1781 | Yes |
| 16 | PRUNE1 | prune exopolyphosphatase 1 [Source:HGNC Symbol;Acc:HGNC:13420] | 691 | 0.596 | 0.1894 | Yes |
| 17 | PAPSS1 | 3'-phosphoadenosine 5'-phosphosulfate synthase 1 [Source:HGNC Symbol;Acc:HGNC:8603] | 703 | 0.593 | 0.2008 | Yes |
| 18 | PDE6D | phosphodiesterase 6D [Source:HGNC Symbol;Acc:HGNC:8788] | 718 | 0.589 | 0.2119 | Yes |
| 19 | CANT1 | calcium activated nucleotidase 1 [Source:HGNC Symbol;Acc:HGNC:19721] | 783 | 0.573 | 0.2187 | Yes |
| 20 | POLD1 | "DNA polymerase delta 1, catalytic subunit [Source:HGNC Symbol;Acc:HGNC:9175]" | 811 | 0.566 | 0.2283 | Yes |
| 21 | POLD4 | "DNA polymerase delta 4, accessory subunit [Source:HGNC Symbol;Acc:HGNC:14106]" | 820 | 0.564 | 0.2393 | Yes |
| 22 | NME3 | NME/NM23 nucleoside diphosphate kinase 3 [Source:HGNC Symbol;Acc:HGNC:7851] | 876 | 0.549 | 0.2464 | Yes |
| 23 | ENTPD1 | ectonucleoside triphosphate diphosphohydrolase 1 [Source:HGNC Symbol;Acc:HGNC:3363] | 909 | 0.539 | 0.2550 | Yes |
| 24 | DCK | deoxycytidine kinase [Source:HGNC Symbol;Acc:HGNC:2704] | 920 | 0.537 | 0.2654 | Yes |
| 25 | PDE1A | phosphodiesterase 1A [Source:HGNC Symbol;Acc:HGNC:8774] | 1074 | 0.500 | 0.2637 | Yes |
| 26 | POLR2K | "RNA polymerase II, I and III subunit K [Source:HGNC Symbol;Acc:HGNC:9198]" | 1090 | 0.499 | 0.2729 | Yes |
| 27 | GMPR | guanosine monophosphate reductase [Source:HGNC Symbol;Acc:HGNC:4376] | 1176 | 0.481 | 0.2761 | Yes |
| 28 | APRT | adenine phosphoribosyltransferase [Source:HGNC Symbol;Acc:HGNC:626] | 1178 | 0.481 | 0.2860 | Yes |
| 29 | NUDT9 | nudix hydrolase 9 [Source:HGNC Symbol;Acc:HGNC:8056] | 1365 | 0.448 | 0.2807 | Yes |
| 30 | ADCY7 | adenylate cyclase 7 [Source:HGNC Symbol;Acc:HGNC:238] | 1408 | 0.442 | 0.2866 | Yes |
| 31 | NME2 | NME/NM23 nucleoside diphosphate kinase 2 [Source:HGNC Symbol;Acc:HGNC:7850] | 1428 | 0.439 | 0.2941 | Yes |
| 32 | POLR3C | RNA polymerase III subunit C [Source:HGNC Symbol;Acc:HGNC:30076] | 1504 | 0.429 | 0.2971 | Yes |
| 33 | ADCY1 | adenylate cyclase 1 [Source:HGNC Symbol;Acc:HGNC:232] | 1589 | 0.415 | 0.2991 | Yes |
| 34 | GUK1 | guanylate kinase 1 [Source:HGNC Symbol;Acc:HGNC:4693] | 1640 | 0.407 | 0.3036 | Yes |
| 35 | POLR2E | "RNA polymerase II, I and III subunit E [Source:HGNC Symbol;Acc:HGNC:9192]" | 1760 | 0.389 | 0.3024 | Yes |
| 36 | AMPD1 | adenosine monophosphate deaminase 1 [Source:HGNC Symbol;Acc:HGNC:468] | 1789 | 0.385 | 0.3081 | Yes |
| 37 | NT5C | "5', 3'-nucleotidase, cytosolic [Source:HGNC Symbol;Acc:HGNC:17144]" | 1852 | 0.379 | 0.3111 | Yes |
| 38 | POLR3B | RNA polymerase III subunit B [Source:HGNC Symbol;Acc:HGNC:30348] | 2220 | 0.336 | 0.2893 | No |
| 39 | POLA1 | "DNA polymerase alpha 1, catalytic subunit [Source:HGNC Symbol;Acc:HGNC:9173]" | 2300 | 0.327 | 0.2899 | No |
| 40 | FHIT | fragile histidine triad diadenosine triphosphatase [Source:HGNC Symbol;Acc:HGNC:3701] | 2758 | 0.274 | 0.2598 | No |
| 41 | GUCY2D | "guanylate cyclase 2D, retinal [Source:HGNC Symbol;Acc:HGNC:4689]" | 2832 | 0.267 | 0.2596 | No |
| 42 | IMPDH2 | inosine monophosphate dehydrogenase 2 [Source:HGNC Symbol;Acc:HGNC:6053] | 2867 | 0.264 | 0.2623 | No |
| 43 | POLR2C | RNA polymerase II subunit C [Source:HGNC Symbol;Acc:HGNC:9189] | 2882 | 0.262 | 0.2667 | No |
| 44 | GUCY2C | guanylate cyclase 2C [Source:HGNC Symbol;Acc:HGNC:4688] | 2982 | 0.252 | 0.2641 | No |
| 45 | PDE11A | phosphodiesterase 11A [Source:HGNC Symbol;Acc:HGNC:8773] | 3090 | 0.241 | 0.2607 | No |
| 46 | PRIM1 | DNA primase subunit 1 [Source:HGNC Symbol;Acc:HGNC:9369] | 3241 | 0.227 | 0.2537 | No |
| 47 | ENPP1 | ectonucleotide pyrophosphatase/phosphodiesterase 1 [Source:HGNC Symbol;Acc:HGNC:3356] | 3392 | 0.212 | 0.2463 | No |
| 48 | POLA2 | "DNA polymerase alpha 2, accessory subunit [Source:HGNC Symbol;Acc:HGNC:30073]" | 3494 | 0.203 | 0.2426 | No |
| 49 | RRM2 | ribonucleotide reductase regulatory subunit M2 [Source:HGNC Symbol;Acc:HGNC:10452] | 3505 | 0.202 | 0.2460 | No |
| 50 | NT5E | 5'-nucleotidase ecto [Source:HGNC Symbol;Acc:HGNC:8021] | 3515 | 0.201 | 0.2494 | No |
| 51 | PDE7B | phosphodiesterase 7B [Source:HGNC Symbol;Acc:HGNC:8792] | 3647 | 0.190 | 0.2431 | No |
| 52 | PRPS1 | phosphoribosyl pyrophosphate synthetase 1 [Source:HGNC Symbol;Acc:HGNC:9462] | 3717 | 0.184 | 0.2415 | No |
| 53 | ITPA | inosine triphosphatase [Source:HGNC Symbol;Acc:HGNC:6176] | 3751 | 0.181 | 0.2426 | No |
| 54 | AK5 | adenylate kinase 5 [Source:HGNC Symbol;Acc:HGNC:365] | 3841 | 0.172 | 0.2392 | No |
| 55 | POLE3 | "DNA polymerase epsilon 3, accessory subunit [Source:HGNC Symbol;Acc:HGNC:13546]" | 3934 | 0.162 | 0.2354 | No |
| 56 | GUCY1B1 | guanylate cyclase 1 soluble subunit beta 1 [Source:HGNC Symbol;Acc:HGNC:4687] | 3962 | 0.159 | 0.2365 | No |
| 57 | PKM | pyruvate kinase M1/2 [Source:HGNC Symbol;Acc:HGNC:9021] | 3963 | 0.159 | 0.2398 | No |
| 58 | AMPD3 | adenosine monophosphate deaminase 3 [Source:HGNC Symbol;Acc:HGNC:470] | 4174 | 0.140 | 0.2263 | No |
| 59 | AK2 | adenylate kinase 2 [Source:HGNC Symbol;Acc:HGNC:362] | 4179 | 0.140 | 0.2288 | No |
| 60 | POLE2 | "DNA polymerase epsilon 2, accessory subunit [Source:HGNC Symbol;Acc:HGNC:9178]" | 4237 | 0.134 | 0.2271 | No |
| 61 | NME6 | NME/NM23 nucleoside diphosphate kinase 6 [Source:HGNC Symbol;Acc:HGNC:20567] | 4257 | 0.132 | 0.2284 | No |
| 62 | PRPS2 | phosphoribosyl pyrophosphate synthetase 2 [Source:HGNC Symbol;Acc:HGNC:9465] | 4365 | 0.123 | 0.2226 | No |
| 63 | PRIM2 | DNA primase subunit 2 [Source:HGNC Symbol;Acc:HGNC:9370] | 4491 | 0.114 | 0.2151 | No |
| 64 | GMPS | guanine monophosphate synthase [Source:HGNC Symbol;Acc:HGNC:4378] | 4498 | 0.113 | 0.2170 | No |
| 65 | RRM1 | ribonucleotide reductase catalytic subunit M1 [Source:HGNC Symbol;Acc:HGNC:10451] | 4501 | 0.113 | 0.2192 | No |
| 66 | NUDT2 | nudix hydrolase 2 [Source:HGNC Symbol;Acc:HGNC:8049] | 4521 | 0.112 | 0.2200 | No |
| 67 | ADPRM | "ADP-ribose/CDP-alcohol diphosphatase, manganese dependent [Source:HGNC Symbol;Acc:HGNC:30925]" | 4653 | 0.100 | 0.2118 | No |
| 68 | ADCY9 | adenylate cyclase 9 [Source:HGNC Symbol;Acc:HGNC:240] | 5191 | 0.057 | 0.1709 | No |
| 69 | ADSL | adenylosuccinate lyase [Source:HGNC Symbol;Acc:HGNC:291] | 5685 | 0.017 | 0.1326 | No |
| 70 | ENTPD3 | ectonucleoside triphosphate diphosphohydrolase 3 [Source:HGNC Symbol;Acc:HGNC:3365] | 6052 | -0.008 | 0.1041 | No |
| 71 | PDE3B | phosphodiesterase 3B [Source:HGNC Symbol;Acc:HGNC:8779] | 6077 | -0.010 | 0.1025 | No |
| 72 | POLD2 | "DNA polymerase delta 2, accessory subunit [Source:HGNC Symbol;Acc:HGNC:9176]" | 6311 | -0.028 | 0.0848 | No |
| 73 | NME1 | NME/NM23 nucleoside diphosphate kinase 1 [Source:HGNC Symbol;Acc:HGNC:7849] | 6328 | -0.031 | 0.0842 | No |
| 74 | POLR2B | RNA polymerase II subunit B [Source:HGNC Symbol;Acc:HGNC:9188] | 6426 | -0.037 | 0.0773 | No |
| 75 | GUCY2F | "guanylate cyclase 2F, retinal [Source:HGNC Symbol;Acc:HGNC:4691]" | 6510 | -0.043 | 0.0717 | No |
| 76 | ADCY8 | adenylate cyclase 8 [Source:HGNC Symbol;Acc:HGNC:239] | 6646 | -0.053 | 0.0622 | No |
| 77 | GUCY1A2 | guanylate cyclase 1 soluble subunit alpha 2 [Source:HGNC Symbol;Acc:HGNC:4684] | 6731 | -0.058 | 0.0569 | No |
| 78 | NPR2 | natriuretic peptide receptor 2 [Source:HGNC Symbol;Acc:HGNC:7944] | 6771 | -0.061 | 0.0551 | No |
| 79 | PDE1C | phosphodiesterase 1C [Source:HGNC Symbol;Acc:HGNC:8776] | 6819 | -0.064 | 0.0527 | No |
| 80 | PDE6H | phosphodiesterase 6H [Source:HGNC Symbol;Acc:HGNC:8790] | 6877 | -0.069 | 0.0497 | No |
| 81 | HPRT1 | hypoxanthine phosphoribosyltransferase 1 [Source:HGNC Symbol;Acc:HGNC:5157] | 6954 | -0.076 | 0.0453 | No |
| 82 | ADCY10 | adenylate cyclase 10 [Source:HGNC Symbol;Acc:HGNC:21285] | 7096 | -0.087 | 0.0361 | No |
| 83 | POLR2A | RNA polymerase II subunit A [Source:HGNC Symbol;Acc:HGNC:9187] | 7103 | -0.088 | 0.0374 | No |
| 84 | POLD3 | "DNA polymerase delta 3, accessory subunit [Source:HGNC Symbol;Acc:HGNC:20932]" | 7568 | -0.125 | 0.0037 | No |
| 85 | POLR1D | RNA polymerase I and III subunit D [Source:HGNC Symbol;Acc:HGNC:20422] | 7611 | -0.128 | 0.0030 | No |
| 86 | ENTPD6 | ectonucleoside triphosphate diphosphohydrolase 6 [Source:HGNC Symbol;Acc:HGNC:3368] | 7619 | -0.129 | 0.0051 | No |
| 87 | POLR2D | RNA polymerase II subunit D [Source:HGNC Symbol;Acc:HGNC:9191] | 7726 | -0.136 | -0.0004 | No |
| 88 | NT5M | "5',3'-nucleotidase, mitochondrial [Source:HGNC Symbol;Acc:HGNC:15769]" | 7781 | -0.140 | -0.0017 | No |
| 89 | ADA | adenosine deaminase [Source:HGNC Symbol;Acc:HGNC:186] | 8105 | -0.163 | -0.0236 | No |
| 90 | XDH | xanthine dehydrogenase [Source:HGNC Symbol;Acc:HGNC:12805] | 8366 | -0.183 | -0.0402 | No |
| 91 | PRPS1L1 | phosphoribosyl pyrophosphate synthetase 1 like 1 [Source:HGNC Symbol;Acc:HGNC:9463] | 8382 | -0.184 | -0.0376 | No |
| 92 | ALLC | allantoicase [Source:HGNC Symbol;Acc:HGNC:17377] | 8426 | -0.187 | -0.0371 | No |
| 93 | PDE10A | phosphodiesterase 10A [Source:HGNC Symbol;Acc:HGNC:8772] | 8477 | -0.192 | -0.0370 | No |
| 94 | ADCY3 | adenylate cyclase 3 [Source:HGNC Symbol;Acc:HGNC:234] | 8572 | -0.199 | -0.0403 | No |
| 95 | PDE4D | phosphodiesterase 4D [Source:HGNC Symbol;Acc:HGNC:8783] | 8797 | -0.216 | -0.0534 | No |
| 96 | PDE2A | phosphodiesterase 2A [Source:HGNC Symbol;Acc:HGNC:8777] | 8872 | -0.223 | -0.0545 | No |
| 97 | PDE1B | phosphodiesterase 1B [Source:HGNC Symbol;Acc:HGNC:8775] | 9159 | -0.245 | -0.0719 | No |
| 98 | POLR3F | RNA polymerase III subunit F [Source:HGNC Symbol;Acc:HGNC:15763] | 9229 | -0.250 | -0.0721 | No |
| 99 | POLR2F | "RNA polymerase II, I and III subunit F [Source:HGNC Symbol;Acc:HGNC:9193]" | 9352 | -0.259 | -0.0763 | No |
| 100 | POLR1C | RNA polymerase I and III subunit C [Source:HGNC Symbol;Acc:HGNC:20194] | 9405 | -0.265 | -0.0749 | No |
| 101 | ENTPD5 | ectonucleoside triphosphate diphosphohydrolase 5 (inactive) [Source:HGNC Symbol;Acc:HGNC:3367] | 9414 | -0.265 | -0.0701 | No |
| 102 | GUCY1A1 | guanylate cyclase 1 soluble subunit alpha 1 [Source:HGNC Symbol;Acc:HGNC:4685] | 9420 | -0.266 | -0.0649 | No |
| 103 | PAICS | phosphoribosylaminoimidazole carboxylase and phosphoribosylaminoimidazolesuccinocarboxamide synthase [Source:HGNC Symbol;Acc:HGNC:8587] | 9475 | -0.272 | -0.0636 | No |
| 104 | AMPD2 | adenosine monophosphate deaminase 2 [Source:HGNC Symbol;Acc:HGNC:469] | 9530 | -0.276 | -0.0621 | No |
| 105 | NT5C2 | "5'-nucleotidase, cytosolic II [Source:HGNC Symbol;Acc:HGNC:8022]" | 9595 | -0.283 | -0.0612 | No |
| 106 | PDE6C | phosphodiesterase 6C [Source:HGNC Symbol;Acc:HGNC:8787] | 9633 | -0.286 | -0.0582 | No |
| 107 | NPR1 | natriuretic peptide receptor 1 [Source:HGNC Symbol;Acc:HGNC:7943] | 9634 | -0.286 | -0.0523 | No |
| 108 | PNP | purine nucleoside phosphorylase [Source:HGNC Symbol;Acc:HGNC:7892] | 9657 | -0.288 | -0.0481 | No |
| 109 | AK4 | adenylate kinase 4 [Source:HGNC Symbol;Acc:HGNC:363] | 9682 | -0.290 | -0.0439 | No |
| 110 | ADCY2 | adenylate cyclase 2 [Source:HGNC Symbol;Acc:HGNC:233] | 9715 | -0.293 | -0.0404 | No |
| 111 | GART | "phosphoribosylglycinamide formyltransferase, phosphoribosylglycinamide synthetase, phosphoribosylaminoimidazole synthetase [Source:HGNC Symbol;Acc:HGNC:4163]" | 9861 | -0.307 | -0.0454 | No |
| 112 | PDE4A | phosphodiesterase 4A [Source:HGNC Symbol;Acc:HGNC:8780] | 9957 | -0.317 | -0.0463 | No |
| 113 | POLR3G | RNA polymerase III subunit G [Source:HGNC Symbol;Acc:HGNC:30075] | 10018 | -0.323 | -0.0443 | No |
| 114 | PKLR | pyruvate kinase L/R [Source:HGNC Symbol;Acc:HGNC:9020] | 10120 | -0.333 | -0.0453 | No |
| 115 | PDE5A | phosphodiesterase 5A [Source:HGNC Symbol;Acc:HGNC:8784] | 10441 | -0.366 | -0.0628 | No |
| 116 | PFAS | phosphoribosylformylglycinamidine synthase [Source:HGNC Symbol;Acc:HGNC:8863] | 10943 | -0.424 | -0.0933 | No |
| 117 | IMPDH1 | inosine monophosphate dehydrogenase 1 [Source:HGNC Symbol;Acc:HGNC:6052] | 11023 | -0.433 | -0.0905 | No |
| 118 | POLE | "DNA polymerase epsilon, catalytic subunit [Source:HGNC Symbol;Acc:HGNC:9177]" | 11026 | -0.433 | -0.0817 | No |
| 119 | ENTPD2 | ectonucleoside triphosphate diphosphohydrolase 2 [Source:HGNC Symbol;Acc:HGNC:3364] | 11060 | -0.440 | -0.0752 | No |
| 120 | PDE8B | phosphodiesterase 8B [Source:HGNC Symbol;Acc:HGNC:8794] | 11076 | -0.441 | -0.0673 | No |
| 121 | PDE9A | phosphodiesterase 9A [Source:HGNC Symbol;Acc:HGNC:8795] | 11215 | -0.459 | -0.0686 | No |
| 122 | POLR1E | RNA polymerase I subunit E [Source:HGNC Symbol;Acc:HGNC:17631] | 11274 | -0.468 | -0.0635 | No |
| 123 | PDE6G | phosphodiesterase 6G [Source:HGNC Symbol;Acc:HGNC:8789] | 11275 | -0.468 | -0.0538 | No |
| 124 | PPAT | phosphoribosyl pyrophosphate amidotransferase [Source:HGNC Symbol;Acc:HGNC:9238] | 11534 | -0.505 | -0.0636 | No |
| 125 | PDE3A | phosphodiesterase 3A [Source:HGNC Symbol;Acc:HGNC:8778] | 11546 | -0.507 | -0.0540 | No |
| 126 | PDE8A | phosphodiesterase 8A [Source:HGNC Symbol;Acc:HGNC:8793] | 11831 | -0.555 | -0.0647 | No |
| 127 | ADCY6 | adenylate cyclase 6 [Source:HGNC Symbol;Acc:HGNC:237] | 12005 | -0.594 | -0.0660 | No |
| 128 | PDE4B | phosphodiesterase 4B [Source:HGNC Symbol;Acc:HGNC:8781] | 12057 | -0.609 | -0.0574 | No |
| 129 | POLR2H | "RNA polymerase II, I and III subunit H [Source:HGNC Symbol;Acc:HGNC:9195]" | 12167 | -0.635 | -0.0528 | No |
| 130 | ADSS2 | adenylosuccinate synthase 2 [Source:HGNC Symbol;Acc:HGNC:292] | 12376 | -0.701 | -0.0546 | No |
| 131 | PDE6A | phosphodiesterase 6A [Source:HGNC Symbol;Acc:HGNC:8785] | 12422 | -0.715 | -0.0433 | No |
| 132 | POLR3D | RNA polymerase III subunit D [Source:HGNC Symbol;Acc:HGNC:1080] | 12490 | -0.744 | -0.0332 | No |
| 133 | ENTPD4 | ectonucleoside triphosphate diphosphohydrolase 4 [Source:HGNC Symbol;Acc:HGNC:14573] | 12815 | -0.999 | -0.0379 | No |
| 134 | POLR1B | RNA polymerase I subunit B [Source:HGNC Symbol;Acc:HGNC:20454] | 12841 | -1.045 | -0.0183 | No |
| 135 | PDE4C | phosphodiesterase 4C [Source:HGNC Symbol;Acc:HGNC:8782] | 12861 | -1.103 | 0.0031 | No |
Table: GSEA details [plain text format]

  

Fig 2: KEGG\_PURINE\_METABOLISM      
 Blue-Pink O' Gram in the Space of the Analyzed GeneSet

  

Fig 3: KEGG\_PURINE\_METABOLISM: Random ES distribution      
 Gene set null distribution of ES for **KEGG\_PURINE\_METABOLISM**
